# Health-Related Quality of Life in Primary Sjögren’s Syndrome: Oral Manifestations and Patient-Reported Outcomes

**DOI:** 10.3390/dj14070401

**Published:** 2026-07-02

**Authors:** Sanja Vujović Ristić, Jana Mojsilović, Momir Stevanović, Milica Djurdjević, Marina Kostić, Ana Barjaktarević, Sanja Knežević, Dragan Milovanović

**Affiliations:** 1 Department of Dentistry, Faculty of Medical Sciences, University of Kragujevac, Svetozara Markovića 69, 34000 Kragujevac, Serbia; sanja.994@live.com (S.V.R.); momirstevanovic7@gmail.com (M.S.); milica.vel94@gmail.com (M.D.); 2 Department of Pharmacology and Toxicology, Faculty of Medical Sciences, University of Kragujevac, Svetozara Markovića 69, 34000 Kragujevac, Serbia; marrina2006kg@yahoo.com (M.K.); piki@fmn.kg.ac.rs (D.M.); 3 Center for Research on Harmful Effects of Biological and Chemical Hazards, Faculty of Medical Sciences, University of Kragujevac, 34000 Kragujevac, Serbia; 4 Department of Pharmacy, Faculty of Medical Sciences, University of Kragujevac, 69, 34000 Kragujevac, Serbia; ana.radovanovickg@gmail.com; 5 Department of Pediatrics, Faculty of Medical Sciences, University of Kragujevac, Svetozara Markovića 69, 34000 Kragujevac, Serbia; sanjaknez1980@yahoo.com

**Keywords:** primary Sjögren’s syndrome, health-related quality of life, questionnaires, oral manifestations, xerostomia

## Abstract

**Background/Objectives**: Primary Sjögren’s syndrome (pSS) is a chronic autoimmune rheumatic disease that clinically presents with symptoms of xerostomia and xerophthalmia, as well as a wide range of other symptoms that may affect patients’ daily functioning and life satisfaction. The main purpose of this study was to assess their health-related quality of life (HRQoL) using both general and disease-specific questionnaires. **Methods**: This cross-sectional observational research with prospective data collection was conducted at the Rheumatology Clinic of the University Clinical Centre of Kragujevac. Participants were divided into two groups: patients with oral manifestations (oral manifestations group) and those presenting with xerostomia only, without other oral lesions or symptoms (xerostomia-only group). A complete clinical examination of the patient’s oral cavity was performed by one doctor of dental medicine. HRQoL was evaluated using various generic and disease-specific instruments. **Results**: A total of 80 participants were included in the study, of whom 40 were in the oral manifestations group and 40 in the xerostomia-only group. Patients with oral manifestations had significantly higher scores across all PSS-QoL domains compared with the xerostomia-only group (*p* < 0.001). A statistically significant difference in the total EQ-5D result was detected between groups (0.7 (0.3) vs. 0.8 (0.1), *p* < 0.001). In multivariable regression analysis (R^2^ = 0.921), the ESSPRI score (β = 0.418, *p* < 0.001) and the presence of oral manifestations (β = −1.155, *p* < 0.001) were significant independent predictors of impaired HRQoL, while disease activity showed no significant association (*p* = 0.895). **Conclusions**: Patients with primary Sjögren’s syndrome presenting with oral manifestations have poorer HRQoL compared with participants with xerostomia only. Symptom burden, including dryness, pain, fatigue, and oral manifestations, may be associated with decreased HRQoL, in contrast to disease activity.

## 1. Introduction

Primary Sjögren’s syndrome (pSS) is a chronic autoimmune rheumatic disease that clinically presents with symptoms of xerostomia and xerophthalmia due to the salivary and lacrimal glands’ lymphocytic infiltration [1]. It is not a rare condition considering its prevalence of 0.5 to 1% in the population, with a marked preference for middle-aged females [2,3]. Approximately half of the pSS patients, alongside profound dryness and chronic pain, develop various extraglandular complications, including fatigue, arthritis, skin vasculitis, peripheral neuropathy, glomerular nephritis, and mental disorders such as anxiety and depression [4]. Currently, there are no effective disease-modifying treatment modalities for pSS, making it an area of significant unmet medical need [5,6]. It is well established that physical, psychological, and social factors are fundamental to an individual’s perceived well-being; therefore, oral, ocular, and systemic manifestations of the pSS might substantially impair patients’ overall quality of life (QoL) [2,7].

Health-related quality of life (HRQoL) determines how different health conditions and therapeutic approaches impact the functioning of patients, encompassing simultaneously physical, psychological, and social aspects [6]. It has been recognized as one of the essential outcomes of clinical research. HRQoL is commonly assessed by multiple generic and disease-specific questionnaires designed to measure the effect of health status on the various domains of the QoL [8]. Additionally, this concept has a vital role in medical decision-making and health economic evaluations [5,6]. The SF-36 Health Survey (SF-36) and EuroQoL-5D (EQ-5D) are the most frequently used instruments in studies evaluating the HRQoL in patients with pSS. Their results consistently demonstrate a notable decline in their HRQoL compared to healthy controls, which is mostly attributed to sicca symptoms, chronic pain, and fatigue [3,9,10,11]. Recently, a focus has been put on disease-specific tools as they effectively highlight the key elements of disease, therefore providing a more thorough understanding of a patient’s perspective. So far, only one instrument has been developed for assessment of the HRQoL in pSS, named PSS-QoL questionnaire, which addresses both the physical and psychosocial aspects of this condition [12].

The main purpose of this study is to assess the HRQoL in pSS patients using both general and disease-specific questionnaires. Additionally, the study aims to explore whether oral manifestations, disease activity or patient-reported symptoms (dryness, pain, and fatigue) are predictors of the HRQoL in this population.

## 2. Materials and Methods

### 2.1. Research Design and Participants Recruitment

This cross-sectional observational research with prospective data collection was conducted at the Rheumatology Clinic of the University Clinical Centre of Kragujevac between July 2021 and September 2022. All participants were assessed at a single time point, with no follow-up period. The study protocol was approved by the Ethics Committee of the University Clinical Centre of Kragujevac (decision number 01/20-657). The research was performed in compliance with the Helsinki Declaration of 1964 and its later amendments, as an academic, non-profit project. Eighty patients with the diagnosis of pSS based on the American College of Rheumatology/European League Against Rheumatism (ACR/EULAR) classification criteria, aged over 18, were included in the study [13]. Patients under 18, and those with systemic connective tissue disorders, mental illnesses, oncological conditions, and active nicotine users were excluded. All the participants signed the written informed consent, prior to research onset. Subjects were allocated into two groups according to their oral status. The first one (oral manifestations group) was composed of patients who presented with different manifestations and symptoms in the oral cavity, while the second (xerostomia-only group) included participants who complained only of xerostomia, without other pathological lesions and symptoms detected.

### 2.2. Sample Size

The sample size was estimated a priori using G*Power software (version 3.1.9.7; Heinrich Heine University Düsseldorf, Düsseldorf, Germany), based on effect sizes reported in a previous study by Fernández Castro et al. [14], indicating that at least 40 participants per group would be required.

### 2.3. Clinical Examination of the Oral Cavity

Prior to the medical evaluation, a comprehensive questionnaire regarding sociodemographic and clinical data was administered to all participants. Comorbidities were defined as the presence of chronic systemic conditions, including hypertension, diabetes mellitus, and other long-term diseases. Information on current medication use was obtained from medical records and patient reports. A complete clinical examination of the patient’s oral cavity was performed by one doctor of dental medicine (periodontology and oral medicine resident) at the Department of Dentistry of the Faculty of Medical Sciences, University of Kragujevac. The evaluation of the oral conditions was performed following WHO protocols, using a dental mirror and probe under specialized dental light, with the lesion type, location, clinical presentation, and related symptoms recorded [15]. Oral manifestations comprised exfoliative cheilitis, angular cheilitis, aphthous ulcers, ulcerations, gingivitis, caries in atypical locations, geographic tongue, and oral fungal infections. Reported oral symptoms included difficulty chewing, speaking, and swallowing, burning sensations in the oral cavity, and difficulty wearing dentures. According to the findings of the oral examination, participants were categorized into two groups, as previously mentioned.

### 2.4. Evaluation of the Health-Related Quality of Life

#### 2.4.1. Generic HRQoL Instruments

SF-36 is a widely applied generic instrument that consists of 8 domains: physical functioning, role limitations due to physical problems, bodily pain, general health, vitality, social functioning, role limitations due to emotional problems, and mental health. Scores range from 0, indicating the worst possible health to 100, reflecting the best possible health [16]. EQ-5D represents a standardized HRQoL measure, available in numerous languages, designed to evaluate health across a broad spectrum of conditions and diseases. It includes a brief five-dimension questionnaire (mobility, self-care, usual activities, pain/discomfort, anxiety/depression) that describes an individual’s health status, along with a visual analog scale (EQ-VAS) that captures the respondent’s perception of their current health on a numeric scale from 0 (the worst health you can imagine) to 100 (the best health you can imagine). The total score is derived using country-specific value sets [17].

#### 2.4.2. Disease-Specific Instruments

FACIT-Fatigue is a 13-item tool created to evaluate the severity of fatigue and its effect on the daily functioning of patients with various chronic illnesses, including pSS. Responses are recorded on a 5-point Likert scale, ranging from “not at all” to “very much.” The maximum possible result is 52, with higher values implicating lower levels of fatigue and better overall HRQoL [18]. PSS-QoL is the first instrument developed specifically for the assessment of HRQoL in pSS patients. It comprises physical (discomfort—6 questions—and dryness—5 questions) and psychosocial (14 questions) domains. The overall score is computed by summing the individual item responses and it might range from 0 to 96 for females and 0 to 92 for males (excluding the vaginal dryness item). Higher results suggest poorer HRQoL of pSS patients [12,19].

### 2.5. Disease Activity

Disease activity was determined by applying the EULAR SS Disease Activity Index (ESSDAI) and EULAR SS Patient-Reported Index (ESSPRI). ESSDAI, developed in 2009 through collaborative efforts of European and American researchers, is regarded as a gold standard for assessing the disease activity in pSS patients. It consists of 12 domains, each categorized into 3 to 4 levels of activity, corresponding to specific organ systems (e.g., glandular, cutaneous, renal…). It has been demonstrated that ESSDAI can be applied in both clinical trials and daily practice due to its sensitivity in discerning fluctuations in disease activity levels and its high reproducibility [20]. ESSDAI score may range from 0 to 123, with values lower than 5 reflecting low disease activity, values between 5 and 13 pointing to moderate disease activity, and values of 14 and greater suggesting high disease activity [21]. The key symptoms related to pSS (dryness, fatigue, and pain) were evaluated using ESSPRI, a self-administered instrument, designed in 2011, that comprises 3 numeric scales (ranging from 0, indicating no dryness/fatigue/pain to 10, implying maximal imaginable dryness/fatigue/pain in the past two weeks). The overall result is derived as the mean of the three domain scores. A value of less than 5 demonstrates a satisfactory state from the patient’s perspective, whereas a value of 5 or greater signifies an unsatisfactory state [21,22]. It is recommended to implement both of these indexes simultaneously to obtain a comprehensive assessment of the patient’s disease activity and symptom burden.

### 2.6. Statistical Analysis

Data analysis was conducted using the SPSS statistical program, version 22.0 (IBM Corp., Armonk, NY, USA) and Jamovi software (version 2.7.32; The jamovi project). Categorical variables were presented as frequencies and percentages. The normality of continuous variables was assessed using the Kolmogorov–Smirnov test. Since the data displayed a non-normal distribution, continuous variables were expressed as the median and interquartile range (IQR). For the comparison of categorical variables, the Chi-square test or Fisher’s exact test was applied, as appropriate. The Mann–Whitney U test was performed for the comparison of numerical variables between the two groups. Spearman’s rank correlation test was utilized to evaluate the level of association between variables of interest. A multivariable linear regression analysis was conducted to determine the independent contribution of oral manifestations, disease activity, and patient-reported symptoms to the HRQoL. Multicollinearity among the predictors in the regression model was assessed using the Variance Inflation Factor (VIF). A *p*-value < 0.05 was deemed statistically significant. Graphical representations of the data were created using Microsoft Excel 2021 (Microsoft Corporation, Redmond, WA, USA) and Jamovi (version 2.7.32; The jamovi project).

## 3. Results

### 3.1. Participants’ Characteristics

Eighty patients diagnosed with pSS were included in the research. The majority of the participants were females (96.3%). The median age of the study subjects was 65.5 years, with a range from 26 to 81 years. There were no significant differences between groups regarding their sociodemographic characteristics (*p* > 0.05). However, statistically significant differences were observed in the median age at disease onset (52.0 years in the oral manifestations group vs. 58.0 years in the xerostomia-only group; *p* < 0.05) and disease duration (10.0 years in the oral manifestations group vs. 7.0 years in the xerostomia-only group; *p* < 0.05). Participants’ sociodemographic characteristics are depicted in Table 1. The clinical data for pSS are summarized in Table 2, with the only statistically significant difference between the groups noted in the presence of systemic complications related to pSS (*p* < 0.05).

### 3.2. Health-Related Quality of Life

#### 3.2.1. Generic HRQoL Instruments

Table 3 shows the SF-36 results, revealing that pSS patients with oral manifestations exhibit significantly lower values across all measured domains.

Table 4 displays the frequency and percentage distributions of responses for each EQ-5D dimension. The majority of the patients reported no problems with mobility (73.7%), self-care (81.2%), or usual activities (67.5%). However, 62.5% of the subjects with oral manifestations recorded a moderate level of pain/discomfort, while 45.0% experienced moderate mental health issues.

A statistically significant difference in the total EQ-5D score was detected between the oral manifestations and xerostomia-only groups (0.7 (0.3) vs. 0.8 (0.1); *p* < 0.001), indicating that pSS patients with oral manifestations have a diminished HRQoL compared to subjects without such lesions and symptoms.

**Figure 1:**
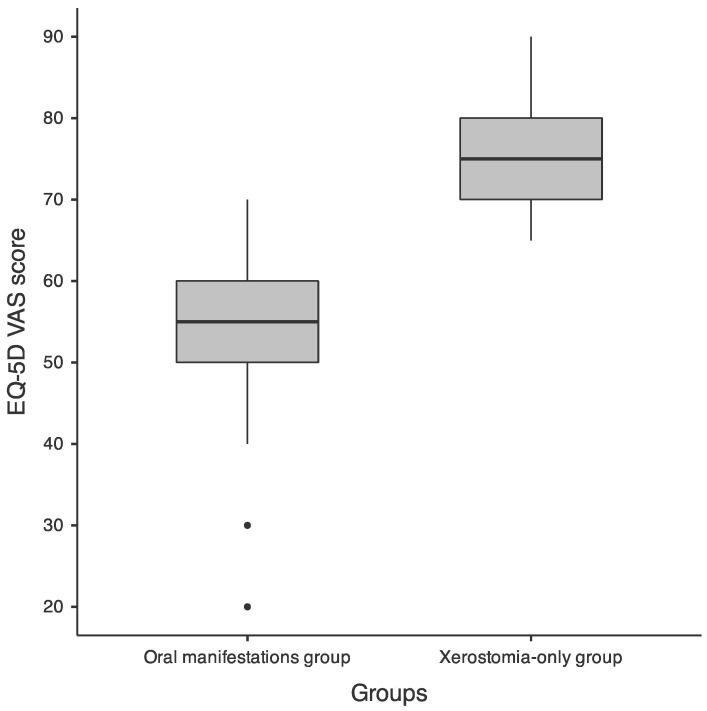
EQ-VAS.

Figure 1 shows the results of the EQ-VAS evaluation. A statistically significant difference between the groups was found regarding the participants’ perspective on their current health status (55.0 (10.0) vs. 75.0 (10.0); *p* < 0.001).

#### 3.2.2. Disease-Specific Instruments

**Figure 2:**
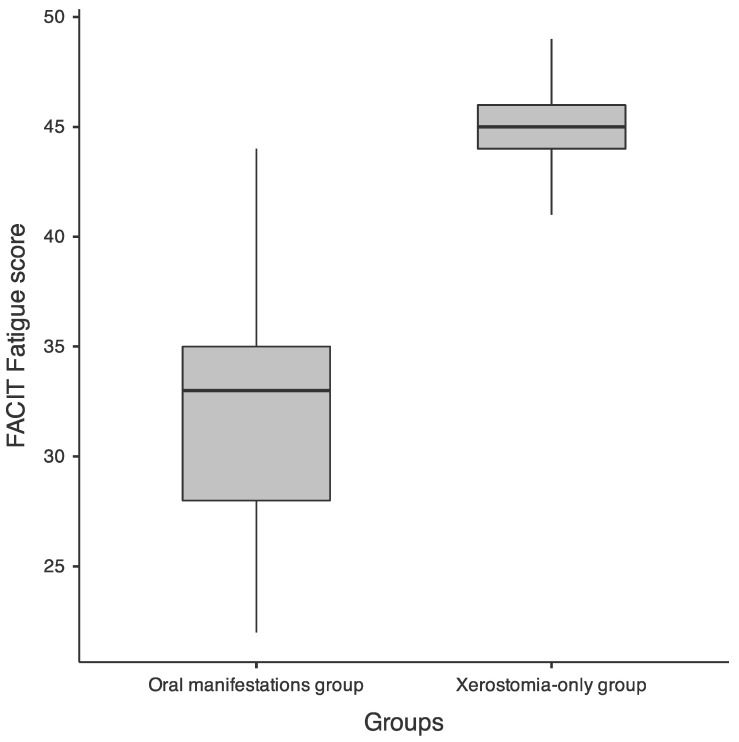
FACIT-Fatigue.

Figure 2 illustrates the outcomes of the FACIT-Fatigue scale analysis. The data show that pSS patients with oral manifestations report lower median values (33.0 (7.0) vs. 45.0 (2.0); *p* < 0.001), suggesting that fatigue significantly impacts their daily functioning and HRQoL.

**Figure 3:**
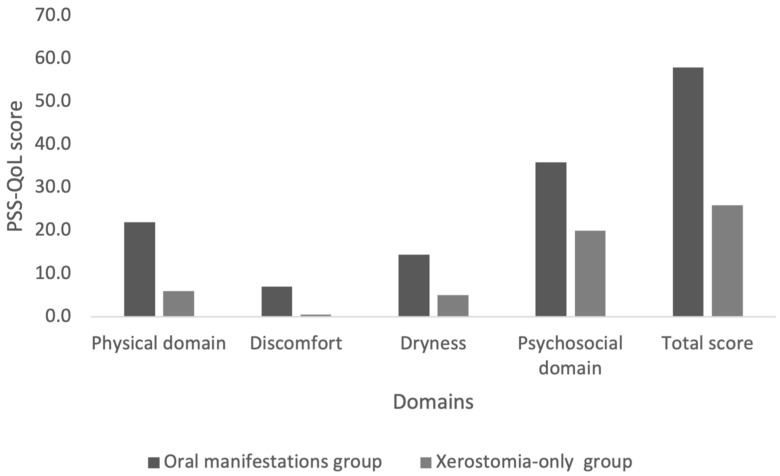
PSS-QoL.

Figure 3 provides a graphical representation of the PSS-QoL scale results. Subjects with pSS experiencing oral manifestations record higher scores across all domains (*p* < 0.001), implying that oral complications and symptoms are associated with a decreased HRQoL in this population.

### 3.3. Disease Activity

Table 5 presents the distribution of participants according to their ESSDAI and ESSPRI scores.

Table 6 displays the median values of the ESSDAI. A statistically significant difference was observed between the groups (*p* < 0.05).

Table 7 shows the median values of the ESSPRI questionnaire and its subscales. A statistically significant difference was found between the groups across all parameters (*p* < 0.05).

### 3.4. Factors Associated with HRQoL

A multivariable linear regression analysis was conducted to examine the specific impact of disease activity, patient-reported symptoms, and oral manifestations on the HRQoL in pSS patients (Table 8). The model included ESSDAI, ESSPRI, and oral manifestations (absent vs. present) as predictors, with the total PSS-QoL score as the dependent variable. Overall, the model was statistically highly significant (*p* < 0.001) and explained 92.1% of the total variance (R^2^ = 0.921, Adjusted R^2^ = 0.918). Multicollinearity was not a concern, as VIF values ranged from 1.20 to 3.81 (all below the common threshold of 5). In this adjusted model, ESSDAI was not found to be a significant predictor of the HRQoL in pSS patients (*p* = 0.895). In contrast, ESSPRI proved to be a strong independent predictor, with higher ESSPRI scores associated with poorer HRQoL (*p* < 0.001). Furthermore, the presence of oral manifestations significantly predicted worse HRQoL outcomes (*p* < 0.001).

## 4. Discussion

The main objective of this research was to evaluate the HRQoL in pSS patients using both general and disease-specific questionnaires. In addition, the study aimed to determine whether oral manifestations, disease activity, and patient-reported symptoms (dryness, pain, and fatigue) could predict the HRQoL of this population. Our results indicated that their HRQoL is substantially impaired, particularly in patients with oral symptoms. Additionally, key pSS-related symptoms and oral manifestations were significantly associated with HRQoL in the regression model.

HRQoL represents a multidimensional concept that illustrates how various factors such as physical health, psychological state, level of independence, social relationships, and personal beliefs, may be affected by a disease or its treatment. It is commonly used in clinical research to evaluate the overall effect of health conditions beyond objective clinical measures, focusing on the patient’s perceived daily functioning and life satisfaction [23]. The most frequently applied tools in this field are SF-36 and EQ-5D, which are proven to possess good reliability and validity across different health conditions and populations. Our findings showed, based on SF-36 and EQ-5D scores, that pSS patients with oral manifestations have a substantially decreased HRQoL compared with participants without them. Other authors obtained similar results. In one cross-sectional study, it was noted that HRQoL was significantly impaired in pSS patients, due to oral distress being a great burden, with the role emotional domain being the most affected [24]. Similarly, in our research, general health and role emotional are also greatly impacted, which may be explained by the fact that oral health may influence to a great extent an individual’s emotional and social life. The results of a recent cross-sectional study showed that pSS patients have poorer HRQoL when compared to healthy individuals of the same age and gender, which was correlated with a decline in oral health [25]. Pain and vitality were the most affected domains, in contrast to our findings, but these discrepancies may be attributed to differences in disease perception, psychological burden, and clinical phenotype across study populations. Another report indicated that oral health may significantly impact HRQoL in pSS patients, based on SF-36 results, which highlights the importance of timely dental management of the oral manifestations [26]. Similarly, a cross-sectional study that included only female pSS participants showed that both of the SF-36 subscales were substantially affected, with the lowest scores observed in general health and role emotional [27]. These findings align with the outcomes of our research. A British study, conducted on 377 pSS patients, reported EQ-5D scores that differed significantly from those in the general population, indicating impaired HRQoL [5]. A recent study showed that domain pain/discomfort was the most impacted, which is in line with our results. Also, mean values on the EQ-VAS scale were similar to those obtained in this study [28]. Other authors reported that pain and depression were the most important predictors of HRQoL of pSS patients, based on EQ-5D scores [29]. In our work, most of the participants experienced moderate pain and depression, which reflects the impact of pSS on both physical and emotional aspects of an individual’s life. A large cross-sectional study comprising 2961 pSS patients concluded that oral manifestations, particularly mouth ulcers, trouble speaking, and dysphagia, were strongly associated with worse HRQoL, while systemic complications showed a weaker or no significant association with HRQoL [30]. These findings are consistent with our results, emphasizing the important role of oral manifestations in the HRQoL of pSS patients.

PSS-QoL questionnaire is the first disease-specific instrument designed for the evaluation of HRQoL in pSS patients. Our subjects with pSS experiencing oral manifestations recorded higher scores across all domains, implying that oral complications and symptoms are associated with a decreased HRQoL. To date, it has been formally translated into only a few languages, which makes it difficult to compare our results. Still, we find it valuable for both research and routine clinical practice as it might contribute to tailoring individualized therapeutic strategies.

FACIT-Fatigue is a widely used tool to assess fatigue, one of the hallmark symptoms in pSS that affects up to 70% of patients [31]. A recent systematic review confirmed that fatigue is among the most important determinants of impaired HRQoL in pSS patients, supporting our results [32].

Disease activity in pSS is an important clinical parameter that has been investigated in relation to HRQoL, as it may play a role in guiding overall disease management [20]. Our multivariable regression analysis revealed no significant independent contribution of disease activity to HRQoL, which aligns with previous reports in the literature. For instance, a recent cross-sectional study found no significant association between ESSDAI scores and oral health-related quality of life (OHRQoL), evaluated using Oral Health Impact Profile-14 (OHIP-14) questionnaire. In contrast, OHRQoL was strongly related to oral problems, including xerostomia, in pSS patients [33]. Similarly, prior research focusing on functional impairment in pSS demonstrated that limitations in daily activities are more closely linked to patient-reported symptom burden than to systemic disease activity scores [34]. These findings further confirm that patient-perceived well-being and clinical systemic activity represent distinct dimensions of the disease that do not necessarily correlate.

Our study showed that key pSS-associated symptoms and oral manifestations were significant predictors of the HRQoL in pSS patients. A large UK cohort investigation demonstrated that symptom burden significantly predicted decline in HRQoL, which is in line with our findings [5]. Another study showed that pain and fatigue were the main predictors of poor HRQoL, regardless of disease activity [3]. Sicca-related morbidity, fatigue severity, pain, and depression were strongly associated with lower HRQoL in a study that included a large sample of pSS patients [5]. These findings support the concept that symptom burden, rather than disease activity alone, may have a central role in shaping HRQoL in pSS. It also highlights the importance of oral manifestations as an often underrecognized contributor.

The main limitations of this study include the small sample size and the single-center recruitment design. These constraints likely reflect both the low prevalence of pSS in the general population and the fact that Kragujevac has only one tertiary healthcare institution dedicated to the management of systemic autoimmune rheumatic diseases. To address the aforementioned limitations, future multicenter studies involving larger cohorts of patients are necessary to validate and extend our findings. Additionally, potential confounding factors related to xerostomia, such as medication use, were not fully controlled for and may have impacted the observed results. Regarding the statistical analysis, it should be noted that comorbidities, disease duration, and age at disease onset were excluded from the final multivariable regression model. Given that these clinical and chronological factors could independently influence HRQoL, and that duration and onset differed significantly between the examined groups, their exclusion from the model remains a limitation that should be addressed in future longitudinal studies.

## 5. Conclusions

Patients with primary Sjögren’s syndrome presenting with oral manifestations have poorer HRQoL in comparison to participants without them, as demonstrated by both generic and disease-specific questionnaires. In addition, our results indicate that key pSS-related symptoms, including dryness, pain, and fatigue, as well as oral manifestations, are significant predictors of HRQoL in this population. These observations highlight the importance of regular dental care in improving oral health, reducing the burden of oral complications, and enhancing HRQoL. Novel research in this field should primarily focus on identifying strategies to optimize patients’ daily functioning and overall life satisfaction.

## Figures and Tables

**Table 1:** Participants’ sociodemographic characteristics.

Variables ^a^	Oral Manifestations Group *n* (%)	Xerostomia-Only Group *n* (%)	Total *n* (%)	*p*-Value
Gender				1.000
Male	2 (5.0)	1 (2.5)	3 (3.7)	
Female	38 (95.0)	39 (97.5)	77 (96.3)	
Education				0.966
Primary school	9 (22.5)	9 (22.5)	18 (22.5)	
High school	18 (45.0)	20 (50.0)	38 (47.5)	
College	11 (27.5)	10 (25.0)	21 (26.2)	
Master’s/PhD	2 (5.0)	1 (2.5)	3 (3.7)	
Employment				0.789
Yes	4 (10.0)	3 (7.5)	7 (8.7)	
No	12 (30.0)	15 (37.5)	27 (33.7)	
Retired	24 (60.0)	22 (55.0)	46 (57.5)	
Marital status				0.683
Single	1 (2.5)	3 (7.5)	4 (5.0)	
In a relationship	2 (5.0)	3 (7.5)	5 (6.2)	
Married	27 (67.5)	23 (57.5)	50 (62.5)	
Divorced	3 (7.5)	2 (5.0)	5 (6.2)	
Widowed	7 (17.5)	9 (22.5)	16 (20.0)	

^a^ Fisher’s Exact Test.

**Table 2:** Clinical data of pSS patients.

Variables ^a^	Oral Manifestations Group n (%)	Xerostomia-Only Group n (%)	Total n (%)	*p*-Value
**Systemic complications**				0.004
Yes	40 (100.0)	30 (75.0)	70 (87.5)	
No	0 (0.0)	10 (25.0)	10 (12.5)	
**Comorbidities**				0.989
Yes	33 (82.5)	35 (87.5)	68 (85.0)	
No	7 (17.5)	5 (12.5)	12 (15.0)	
**pSS medication**				0.365
Antimalarials	30 (75.0)	32 (80.0)	62 (77.5)	
Antimalarials + corticosteroids	10 (25.0)	8 (20.0)	18 (22.5)	
**Serological status**				
RF+	29 (72.5)	25 (62.5)	54 (67.5)	0.469
ANA+	33 (82.5)	32 (80.0)	65 (81.2)	1.000

pSS—primary Sjögren’s syndrome; RF—rheumatoid factor; ANA—antinuclear antibodies; ^a^ Fisher’s Exact Test.

**Table 3:** SF-36.

SF-36 Domains ^a^	Oral Manifestations Group Median (IQR)	Xerostomia-Only Group Median (IQR)	Total Median (IQR)	*p*-Value
Physical functioning	40.3 (11.5)	51.8 (3.3)	47.9 (13.4)	<0.001
Role physical	36.9 (4.5)	48.2 (6.7)	45.9 (11.2)	<0.001
Bodily pain	38.2 (6.0)	58.8 (10.5)	46.7 (23.8)	<0.001
General health	25.4 (5.5)	29.4 (6.5)	27.0 (7.6)	<0.001
Vitality	37.7 (5.9)	43.7 (2.9)	40.7 (5.9)	<0.001
Social functioning	37.3 (5.0)	47.3 (0.0)	44.8 (10.0)	<0.001
Role emotional	35.3 (13.9)	45.7 (13.9)	45.7 (10.4)	<0.001
Mental health	37.8 (7.8)	49.6 (7.8)	44.3 (13.1)	<0.001

SF-36—Short-Form Health Survey; IQR—interquartile range; ^a^ Mann–Whitney U Test.

**Table 4:** EQ-5D.

Dimension ^a^	Response Options	Oral Manifestations Group n (%)	Xerostomia-Only Group n (%)	Total n (%)	*p*-Value
Mobility	No problems	24 (60.0)	35 (87.5)	59 (73.7)	0.038
	Slight problems	13 (32.5)	5 (12.5)	18 (22.5)	
	Moderate problems	1 (2.5)	0 (0.0)	1 (1.2)	
	Severe problems	1 (2.5)	0 (0.0)	1 (1.2)	
	Unable to walk	1 (2.5)	0 (0.0)	1 (1.2)	
Self-care	No problems	28 (70.0)	37 (92.5)	65 (81.2)	0.057
	Slight problems	9 (22.5)	3 (7.5)	12 (15.0)	
	Moderate problems	1 (2.5)	0 (0.0)	1 (1.2)	
	Severe problems	1 (2.5)	0 (0.0)	1 (1.2)	
	Unable to wash or dress	1 (2.5)	0 (0.0)	1 (1.2)	
Usual activities	No problems	20 (50.0)	34 (85.0)	54 (67.5)	0.003
	Slight problems	15 (37.5)	6 (15.0)	21 (26.2)	
	Moderate problems	3 (7.5)	0 (0.0)	3 (3.7)	
	Severe problems	0 (0.0)	0 (0.0)	0 (0.0)	
	Unable to perform usual activities	2 (5.0)	0 (0.0)	2 (2.5)	
Pain/discomfort	No pain or discomfort	2 (5.0)	23 (57.5)	25 (31.2)	<0.001
	Slight pain or discomfort	2 (5.0)	16 (40.0)	18 (22.5)	
	Moderate pain or discomfort	25 (62.5)	1 (2.5)	26 (32.5)	
	Severe pain or discomfort	11 (27.5)	0 (0.0)	11 (13.7)	
	Extreme pain or discomfort	0 (0.0)	0 (0.0)	0 (0.0)	
Anxiety/depression	Not anxious or depressed	5 (12.5)	14 (35.0)	19 (23.7)	<0.001
	Slightly anxious or depressed	13 (32.5)	24 (60.0)	37 (46.2)	
	Moderately anxious or depressed	18 (45.0)	2 (5.0)	20 (25.0)	
	Severely anxious or depressed	3 (7.5)	0 (0.0)	3 (3.7)	
	Extremely anxious or depressed	1 (2.5)	0 (0.0)	1 (1.2)	

^a^ Fisher’s Exact Test.

**Table 5:** ESSDAI and ESSPRI.

Variables	Oral Manifestations Group *n* (%)	Xerostomia-Only Group *n* (%)	Total *n* (%)	*p*-Value
ESSDAI ^a^				0.003
Low activity	17 (42.5)	28 (70.0)	45 (56.2)	
Moderate activity	21 (52.5)	12 (30.0)	33 (41.2)	
High activity	2 (5.0)	0 (0.0)	2 (2.5)	
ESSPRI ^b^				<0.001
Satisfactory state	2 (5.0)	40 (100.0)	42 (52.5)	
Unsatisfactory state	38 (95.0)	0 (0.0)	38 (47.5)	

ESSDAI—EULAR SS Disease Activity Index; ESSPRI—EULAR SS Patient-Reported Index; ^a^ Chi-square test for trend; ^b^ Fisher’s Exact Test.

**Table 6:** ESSDAI scores.

Variable ^a^	Oral Manifestations Group Median (IQR)	Xerostomia-Only Group Median (IQR)	Total Median (IQR)	*p*-Value
ESSDAI score	6.0 (5.0)	3.0 (3.0)	4.0 (5.0)	0.040

ESSDAI—EULAR SS Disease Activity Index; IQR—interquartile range; ^a^ Mann–Whitney U Test.

**Table 7:** ESSPRI scores.

Variables ^a^	Oral Manifestations Group Median (IQR)	Xerostomia-Only Group Median (IQR)	Total Median (IQR)	*p*-Value
Dryness	6.5 (3.0)	4.0 (2.0)	5.0 (3.0)	<0.001
Fatigue	6.0 (1.0)	1.0 (1.0)	3.5 (5.0)	<0.001
Pain	5.0 (2.0)	0.0 (3.0)	3.0 (5.0)	<0.001
ESSPRI score	6.0 (1.7)	2.3 (1.7)	4.0 (4.0)	<0.001

ESSPRI—EULAR SS Patient-Reported Index; IQR—interquartile range; ^a^ Mann–Whitney U Test.

**Table 8:** Multiple linear regression analysis of factors influencing the HRQoL.

Variables ^a^	B	SE	β	t	95% Confidence Interval		*p*-Value	VIF
					Lower	Upper		
ESSDAI	−0.020	0.151	−0.005	−0.132	−0.321	0.281	0.895	1.20
ESSPRI	3.123	0.470	0.418	6.651	2.188	4.058	<0.001	3.81
Oral manifestations (Absent vs. Present)	−19.066	1.985	−1.155	−9.604	−23.020	−15.112	<0.001	3.53

ESSDAI—EULAR SS Disease Activity Index; ESSPRI—EULAR SS Patient-Reported Index; VIF—Variance Inflation Factor; ^a^ Multivariable linear regression analysis.

## Data Availability

The data presented in this study are available upon request.

## Abbreviations

The following abbreviations are used in this manuscript:

pSS	Primary Sjögren’s Syndrome
QoL	Quality of Life
HRQoL	Health-Related Quality of Life
SF-36	Short-Form-36
EQ-5D	Euro-QoL-5D
PSS-QoL	Primary Sjögren’s Syndrome-Quality of Life
XI	Xerostomia Inventory
ESSDAI	EULAR SS Disease Activity Index
ESSPRI	EULAR SS Patient-Reported Index
